# Penta­carbonyl-1κ^2^ *C*,2κ^3^ *C*-(ferrocenyl­diphenyl­phosphine-1κ*P*)[μ-2-(4-methyl­phen­yl)-2-aza­propane-1,3-dithiol­ato-1:2κ^4^ *S*,*S*′:*S*,*S*′]diiron(I)(*Fe*—*Fe*)

**DOI:** 10.1107/S1600536808032698

**Published:** 2008-10-18

**Authors:** Yan-Feng Tang, Jin-Li Zhu

**Affiliations:** a School of Chemistry and Chemical Engineering, Nantong University, Natong 226007, People’s Republic of China

## Abstract

The title compound, [Fe_2_(C_9_H_11_NS_2_){Fe(C_5_H_5_)(C_17_H_14_P)}(CO)_5_], was prepared as an aza­dithiol­ato–iron model for the iron-only hydrogenase active site. The Fe_2_S_2_ unit exhibits a butterfly conformation and the ferrocenyldiphenyl­phosphine ligand is *trans* to the Fe—Fe bond. The Fe—Fe distance of 2.5160 (8) Å is longer than found in related model structures. Intra­molecular C—H⋯S and inter­molecular C—H⋯O hydrogen bonds are observed.

## Related literature

For general background, see: Cammack (1999 ▶); Evans & Pickett (2003 ▶); Nicolet *et al.* (1999 ▶); Peters *et al.* (1998 ▶). For related structures, see: Hou *et al.* (2006 ▶); Lawrence *et al.* (2001 ▶); Ott *et al.* (2004 ▶).
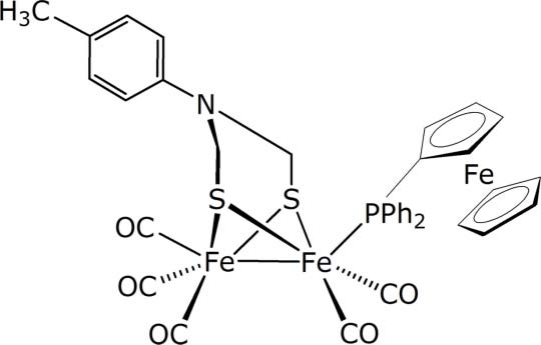


## Experimental

### 

#### Crystal data

[Fe_3_(C_5_H_5_)(C_9_H_11_NS_2_)(C_17_H_14_P)(CO)_5_]
*M* *_r_* = 819.25
Monoclinic, 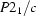

*a* = 13.600 (2) Å
*b* = 10.8964 (19) Å
*c* = 23.396 (4) Å
β = 94.741 (3)°
*V* = 3455.2 (11) Å^3^
*Z* = 4
Mo *K*α radiation
μ = 1.45 mm^−1^
*T* = 298 (2) K
0.20 × 0.20 × 0.10 mm

#### Data collection

Bruker SMART APEX CCD area-detector diffractometer
Absorption correction: multi-scan (*SADABS*; Bruker, 1997 ▶) *T*_min_ = 0.760, *T*_max_ = 0.868
21457 measured reflections
8202 independent reflections
4258 reflections with *I* > 2σ(*I*)
*R*_int_ = 0.070

#### Refinement

*R*[*F* ^2^ > 2σ(*F* ^2^)] = 0.048
*wR*(*F* ^2^) = 0.079
*S* = 0.80
8202 reflections
434 parameters
H-atom parameters constrained
Δρ_max_ = 0.66 e Å^−3^
Δρ_min_ = −0.39 e Å^−3^

### 

Data collection: *SMART* (Bruker, 1997 ▶); cell refinement: *SAINT-Plus* (Bruker, 2001 ▶); data reduction: *SAINT-Plus*; program(s) used to solve structure: *SHELXS97* (Sheldrick, 2008 ▶); program(s) used to refine structure: *SHELXL97* (Sheldrick, 2008 ▶); molecular graphics: *SHELXTL* (Sheldrick, 2008 ▶); software used to prepare material for publication: *SHELXL97*.

## Supplementary Material

Crystal structure: contains datablocks global, I. DOI: 10.1107/S1600536808032698/ci2685sup1.cif

Structure factors: contains datablocks I. DOI: 10.1107/S1600536808032698/ci2685Isup2.hkl

Additional supplementary materials:  crystallographic information; 3D view; checkCIF report

## Figures and Tables

**Table 1:** Selected bond lengths (Å)

Fe1—C13	1.750 (4)
Fe1—P1	2.2565 (10)
Fe1—S1	2.2762 (11)
Fe1—S2	2.2818 (11)
Fe1—Fe2	2.5160 (8)
Fe2—C11	1.761 (5)
Fe2—C12	1.777 (5)
Fe2—C10	1.813 (5)
Fe2—S2	2.2546 (11)
Fe2—S1	2.2867 (11)

**Table 2:** Hydrogen-bond geometry (Å, °)

*D*—H⋯*A*	*D*—H	H⋯*A*	*D*⋯*A*	*D*—H⋯*A*
C22—H22⋯O2^i^	0.98	2.53	3.224 (5)	128
C24—H24⋯S2	0.98	2.72	3.561 (4)	144

Symmetry code: (i) 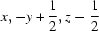.

**Fig. 1.**
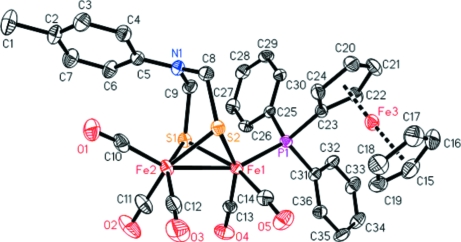
The molecular structure of the title complex, with displacement ellipsoids drawn at the 30% probability level.

## supplementary crystallographic information

### Comment

The iron-only hydrogenases are enzymes that can efficiently catalyze the reversible reaction of hydrogen production (Cammack, 1999; Evans & Pickett, 2003). The active site at which this reaction takes place is composed of a butterfly shaped Fe_2_S_2_ unit covalently linked to a conventional Fe_4_S_4_ cluster by a bridged cysteinyl thioether (Peters *et al.*, 1998; Nicolet *et al.*, 1999). We have prepared the title complex as a structural model for the iron-only hydrogenases active site. Herein we report its crystal structure.

The molecular structure of the title complex is shown in Fig.1 and selected bond distances are listed in Table 1. In the Fe_2_S_2_ unit, the Fe—Fe distance of 2.5160 (8) Å is slightly longer than those observed in related complexes (Lawrence *et al.*, 2001; Ott *et al.*, 2004). The FcPPh_2_ (Fc is ferrocenyl and Ph is phenyl) ligand is in apical position (*trans* to Fe—Fe bond), with an Fe—P distance of 2.2565 (10) Å. The Fe1—C(≡O) distances are ~0.04 Å shorter than those found in a related complex (Hou *et al.*, 2006), consistent with an increase in the charge of Fe1 because one of the CO ligands was replaced by the FcPPh_2_ ligand. The ferrocene unit is covalently linked to the Fe_2_S_2_ unit *via* the bridged diphenyl P atom, with an Fe1—P1—C23 angle of 118.32 (12)°. The N1 atom is displaced from the C5/C8/C9 plane by 0.168 (3) Å, and the sum of angles around N1 is 355.8°.

Intramolecular C—H···S and intermolecular C—H···O hydrogen bonds are observed.

### Experimental

The starting material [Fe_2_(µ-SCH_2_)_2_(4-CH_3_C_6_H_4_N)(CO)_6_] was obtained in 50% yield according to the literature method (Hou *et al.*, 2006). The solution of Me_3_NO (120 mg, 1.06 mmol) was added to a red solution of [Fe_2_(µ-SCH_2_)_2_(4-CH_3_C_6_H_4_N)(CO)_6_] (0.50 g, 1.05 mmol) with FcPPh_2_ (0.39 g, 1.05 mmol) in CH_3_CN (100 ml) *via* syringe under a nitrogen atmosphere. The reaction mixture was stirred at ambient temperature until the TLC indicated there was no remaining carbonyl complex of starting material. The solvent was removed under vacuum and the resulted dark red residue was purified by column chromatography on silica gel eluting with CH_2_Cl_2_-hexane (1:5 *v/v*). A dark red solid was obtained from recrystallization in n-pentane-CH_2_Cl_2_. Single crystals of the title compound for X-ray analysis were grown from a CH_2_Cl_2_-hexane (1:5 *v/v*) solution by slow evaporation at ambient temperature.

### Refinement

H atoms were placed in geometrically calculated positions (C—H = 0.93–0.98 Å) and refined as riding, with *U*_iso_(H) = 1.2*U*_eq_(C) and 1.5*U*_eq_(C_methyl_)

### Crystal data

**Table:**

[Fe_3_(C_5_H_5_)(C_9_H_11_NS_2_)(C_17_H_14_P)(CO)_5_]	*F*(000) = 1672
*M**_r_* = 819.25	*D*_x_ = 1.575 Mg m^−^^3^
Monoclinic, *P*2_1_/*c*	Mo *K*α radiation, λ = 0.71073 Å
Hall symbol: -P 2ybc	Cell parameters from 2549 reflections
*a* = 13.600 (2) Å	θ = 2.4–22.0°
*b* = 10.8964 (19) Å	µ = 1.45 mm^−^^1^
*c* = 23.396 (4) Å	*T* = 298 K
β = 94.741 (3)°	Block, red
*V* = 3455.2 (11) Å^3^	0.20 × 0.20 × 0.10 mm
*Z* = 4	

### Data collection

**Table:**

Bruker SMART APEX CCD area-detector diffractometer	8202 independent reflections
Radiation source: fine-focus sealed tube	4258 reflections with *I* > 2σ(*I*)
graphite	*R*_int_ = 0.070
φ and ω scans	θ_max_ = 28.4°, θ_min_ = 1.5°
Absorption correction: multi-scan (SADABS; Bruker, 1997)	*h* = −17→18
*T*_min_ = 0.760, *T*_max_ = 0.868	*k* = −9→14
21457 measured reflections	*l* = −31→30

### Refinement

**Table:**

Refinement on *F*^2^	0 restraints
Least-squares matrix: full	H-atom parameters constrained
*R*[*F*^2^ > 2σ(*F*^2^)] = 0.048	*w* = 1/[σ^2^(*F*_o_^2^) + (0.0157*P*)^2^] where *P* = (*F*_o_^2^ + 2*F*_c_^2^)/3
*wR*(*F*^2^) = 0.079	(Δ/σ)_max_ = 0.001
*S* = 0.80	Δρ_max_ = 0.66 e Å^−^^3^
8202 reflections	Δρ_min_ = −0.38 e Å^−^^3^
434 parameters	

### Special details

**Table:**

Geometry. All e.s.d.'s (except the e.s.d. in the dihedral angle between two l.s. planes) are estimated using the full covariance matrix. The cell e.s.d.'s are taken into account individually in the estimation of e.s.d.'s in distances, angles and torsion angles; correlations between e.s.d.'s in cell parameters are only used when they are defined by crystal symmetry. An approximate (isotropic) treatment of cell e.s.d.'s is used for estimating e.s.d.'s involving l.s. planes.
Refinement. Refinement of *F*^2^ against ALL reflections. The weighted *R*-factor *wR* and goodness of fit *S* are based on *F*^2^, conventional *R*-factors *R* are based on *F*, with *F* set to zero for negative *F*^2^. The threshold expression of *F*^2^ > σ(*F*^2^) is used only for calculating *R*-factors(gt) *etc*. and is not relevant to the choice of reflections for refinement. *R*-factors based on *F*^2^ are statistically about twice as large as those based on *F*, and *R*- factors based on ALL data will be even larger.

### Fractional atomic coordinates and isotropic or equivalent isotropic displacement parameters (Å^2^)

**Table:**

	*x*	*y*	*z*	*U*_iso_*/*U*_eq_	
Fe1	0.20737 (4)	0.18453 (5)	0.30929 (2)	0.03252 (14)	
Fe2	0.20176 (4)	0.09491 (5)	0.40808 (2)	0.03988 (16)	
Fe3	0.08083 (4)	0.09519 (5)	0.11522 (2)	0.04244 (16)	
S1	0.34466 (7)	0.12772 (9)	0.36528 (4)	0.0387 (2)	
S2	0.15575 (7)	−0.00994 (9)	0.32718 (4)	0.0382 (2)	
P1	0.26239 (7)	0.19707 (9)	0.22109 (4)	0.0316 (2)	
O1	0.2115 (2)	−0.1033 (3)	0.49363 (13)	0.0791 (11)	
O2	0.2652 (3)	0.3021 (3)	0.48103 (14)	0.1006 (13)	
O3	−0.0037 (2)	0.1481 (3)	0.42412 (14)	0.0962 (12)	
O4	0.2502 (2)	0.4345 (2)	0.34576 (12)	0.0634 (9)	
O5	0.0021 (2)	0.2479 (3)	0.27912 (12)	0.0661 (9)	
N1	0.3407 (2)	−0.1259 (3)	0.34951 (12)	0.0409 (8)	
C1	0.4517 (4)	−0.3802 (4)	0.56016 (18)	0.0874 (16)	
H1A	0.4496	−0.4671	0.5535	0.131*	
H1B	0.4066	−0.3592	0.5881	0.131*	
H1C	0.5173	−0.3566	0.5741	0.131*	
C2	0.4224 (3)	−0.3132 (4)	0.50438 (17)	0.0536 (11)	
C3	0.3533 (3)	−0.3595 (4)	0.46376 (18)	0.0529 (11)	
H3	0.3241	−0.4348	0.4706	0.063*	
C4	0.3255 (3)	−0.2992 (4)	0.41345 (16)	0.0459 (10)	
H4	0.2792	−0.3346	0.3870	0.055*	
C5	0.3662 (3)	−0.1853 (4)	0.40172 (16)	0.0394 (9)	
C6	0.4364 (3)	−0.1384 (4)	0.44210 (17)	0.0485 (11)	
H6	0.4658	−0.0634	0.4353	0.058*	
C7	0.4635 (3)	−0.2009 (4)	0.49226 (17)	0.0579 (12)	
H7	0.5107	−0.1667	0.5186	0.069*	
C8	0.2455 (3)	−0.1362 (3)	0.31877 (16)	0.0479 (11)	
H8A	0.2156	−0.2121	0.3304	0.057*	
H8B	0.2551	−0.1436	0.2783	0.057*	
C9	0.3917 (3)	−0.0175 (3)	0.33541 (15)	0.0448 (10)	
H9A	0.3883	−0.0103	0.2940	0.054*	
H9B	0.4606	−0.0266	0.3490	0.054*	
C10	0.2131 (3)	−0.0291 (4)	0.45986 (18)	0.0539 (12)	
C11	0.2391 (3)	0.2194 (4)	0.45270 (18)	0.0651 (14)	
C12	0.0758 (3)	0.1262 (4)	0.41723 (17)	0.0561 (12)	
C13	0.2339 (3)	0.3362 (4)	0.32971 (15)	0.0385 (10)	
C14	0.0838 (3)	0.2218 (3)	0.28947 (16)	0.0431 (10)	
C15	0.0207 (4)	0.2515 (5)	0.0789 (2)	0.0749 (15)	
H15	0.0568	0.3196	0.0630	0.090*	
C16	−0.0165 (4)	0.1463 (5)	0.0488 (2)	0.0738 (15)	
H16	−0.0101	0.1287	0.0081	0.089*	
C17	−0.0633 (3)	0.0727 (5)	0.0861 (2)	0.0736 (15)	
H17	−0.0958	−0.0058	0.0766	0.088*	
C18	−0.0557 (3)	0.1293 (5)	0.1406 (2)	0.0686 (14)	
H18	−0.0826	0.0979	0.1754	0.082*	
C19	−0.0044 (3)	0.2407 (4)	0.1355 (2)	0.0662 (14)	
H19	0.0115	0.2999	0.1664	0.079*	
C20	0.1400 (3)	−0.0774 (4)	0.12177 (17)	0.0518 (11)	
H20	0.1039	−0.1546	0.1152	0.062*	
C21	0.1848 (3)	−0.0097 (4)	0.08032 (18)	0.0551 (12)	
H21	0.1854	−0.0312	0.0397	0.066*	
C22	0.2278 (3)	0.0960 (4)	0.10657 (15)	0.0440 (10)	
H22	0.2638	0.1598	0.0874	0.053*	
C23	0.2103 (2)	0.0944 (3)	0.16603 (14)	0.0331 (9)	
C24	0.1549 (3)	−0.0139 (3)	0.17502 (16)	0.0429 (10)	
H24	0.1310	−0.0401	0.2115	0.051*	
C25	0.3951 (2)	0.1685 (3)	0.22119 (14)	0.0303 (8)	
C26	0.4591 (3)	0.2380 (3)	0.25719 (15)	0.0380 (9)	
H26	0.4338	0.2998	0.2791	0.046*	
C27	0.5593 (3)	0.2175 (4)	0.26120 (16)	0.0471 (11)	
H27	0.6011	0.2650	0.2857	0.056*	
C28	0.5975 (3)	0.1257 (4)	0.22858 (17)	0.0483 (11)	
H28	0.6650	0.1103	0.2315	0.058*	
C29	0.5357 (3)	0.0582 (3)	0.19212 (16)	0.0455 (11)	
H29	0.5618	−0.0015	0.1693	0.055*	
C30	0.4351 (3)	0.0770 (3)	0.18864 (15)	0.0370 (9)	
H30	0.3937	0.0283	0.1645	0.044*	
C31	0.2548 (3)	0.3488 (3)	0.18691 (15)	0.0343 (9)	
C32	0.3189 (3)	0.3814 (3)	0.14616 (16)	0.0477 (11)	
H32	0.3677	0.3266	0.1371	0.057*	
C33	0.3115 (3)	0.4942 (4)	0.11883 (17)	0.0577 (12)	
H33	0.3544	0.5141	0.0913	0.069*	
C34	0.2412 (3)	0.5761 (4)	0.13229 (18)	0.0591 (12)	
H34	0.2364	0.6519	0.1140	0.071*	
C35	0.1777 (3)	0.5472 (4)	0.17249 (18)	0.0547 (12)	
H35	0.1301	0.6034	0.1817	0.066*	
C36	0.1843 (3)	0.4339 (3)	0.19954 (15)	0.0427 (10)	
H36	0.1405	0.4148	0.2267	0.051*	

### Atomic displacement parameters (Å^2^)

**Table:**

	*U*^11^	*U*^22^	*U*^33^	*U*^12^	*U*^13^	*U*^23^
Fe1	0.0317 (3)	0.0340 (3)	0.0323 (3)	−0.0013 (3)	0.0052 (2)	−0.0022 (2)
Fe2	0.0415 (4)	0.0483 (4)	0.0305 (3)	−0.0029 (3)	0.0065 (3)	−0.0030 (3)
Fe3	0.0349 (3)	0.0523 (4)	0.0393 (3)	0.0000 (3)	−0.0020 (3)	−0.0046 (3)
S1	0.0357 (6)	0.0427 (6)	0.0374 (6)	−0.0044 (5)	0.0019 (5)	0.0000 (5)
S2	0.0405 (6)	0.0395 (6)	0.0343 (6)	−0.0058 (5)	0.0014 (5)	0.0002 (4)
P1	0.0296 (5)	0.0336 (6)	0.0319 (6)	0.0006 (5)	0.0038 (4)	−0.0019 (4)
O1	0.103 (3)	0.087 (3)	0.051 (2)	0.012 (2)	0.0248 (19)	0.0228 (18)
O2	0.138 (3)	0.099 (3)	0.067 (3)	−0.033 (3)	0.026 (2)	−0.042 (2)
O3	0.055 (2)	0.151 (4)	0.084 (3)	0.020 (2)	0.018 (2)	−0.018 (2)
O4	0.067 (2)	0.046 (2)	0.079 (2)	−0.0107 (16)	0.0131 (17)	−0.0186 (16)
O5	0.0356 (18)	0.080 (2)	0.083 (2)	0.0090 (16)	0.0092 (17)	0.0150 (17)
N1	0.049 (2)	0.038 (2)	0.036 (2)	0.0013 (16)	0.0032 (17)	0.0055 (15)
C1	0.098 (4)	0.094 (4)	0.067 (4)	−0.003 (3)	−0.013 (3)	0.034 (3)
C2	0.056 (3)	0.057 (3)	0.047 (3)	0.001 (2)	0.002 (2)	0.018 (2)
C3	0.055 (3)	0.047 (3)	0.058 (3)	−0.009 (2)	0.010 (2)	0.012 (2)
C4	0.049 (3)	0.044 (3)	0.043 (3)	−0.006 (2)	−0.002 (2)	−0.001 (2)
C5	0.041 (2)	0.040 (3)	0.037 (2)	0.004 (2)	0.0059 (19)	0.0015 (19)
C6	0.042 (3)	0.049 (3)	0.054 (3)	−0.007 (2)	−0.001 (2)	0.014 (2)
C7	0.050 (3)	0.062 (3)	0.059 (3)	−0.004 (2)	−0.011 (2)	0.008 (2)
C8	0.063 (3)	0.035 (3)	0.044 (3)	0.001 (2)	−0.006 (2)	−0.0051 (19)
C9	0.044 (2)	0.046 (3)	0.046 (3)	0.012 (2)	0.014 (2)	0.009 (2)
C10	0.045 (3)	0.080 (4)	0.039 (3)	0.002 (2)	0.016 (2)	−0.006 (2)
C11	0.072 (3)	0.083 (4)	0.043 (3)	−0.010 (3)	0.022 (3)	−0.013 (3)
C12	0.056 (3)	0.074 (3)	0.039 (3)	0.002 (3)	0.005 (2)	−0.006 (2)
C13	0.032 (2)	0.044 (3)	0.040 (2)	0.004 (2)	0.0056 (18)	−0.004 (2)
C14	0.045 (3)	0.037 (3)	0.050 (3)	−0.006 (2)	0.012 (2)	0.0042 (19)
C15	0.073 (4)	0.067 (4)	0.082 (4)	0.013 (3)	−0.011 (3)	0.016 (3)
C16	0.065 (4)	0.094 (5)	0.058 (3)	0.017 (3)	−0.021 (3)	−0.009 (3)
C17	0.038 (3)	0.087 (4)	0.093 (4)	−0.001 (3)	−0.016 (3)	−0.020 (3)
C18	0.035 (3)	0.098 (4)	0.073 (4)	0.004 (3)	0.005 (2)	−0.013 (3)
C19	0.045 (3)	0.071 (4)	0.080 (4)	0.020 (3)	−0.012 (3)	−0.021 (3)
C20	0.055 (3)	0.042 (3)	0.056 (3)	−0.001 (2)	−0.011 (2)	−0.013 (2)
C21	0.047 (3)	0.076 (3)	0.042 (3)	0.003 (2)	0.002 (2)	−0.024 (2)
C22	0.038 (2)	0.058 (3)	0.036 (2)	−0.005 (2)	0.0070 (19)	−0.006 (2)
C23	0.030 (2)	0.037 (2)	0.032 (2)	0.0003 (18)	0.0013 (17)	−0.0005 (18)
C24	0.046 (3)	0.045 (3)	0.036 (2)	−0.002 (2)	−0.001 (2)	−0.0014 (19)
C25	0.030 (2)	0.032 (2)	0.030 (2)	0.0019 (17)	0.0070 (17)	0.0068 (16)
C26	0.037 (2)	0.036 (2)	0.042 (2)	−0.0003 (19)	0.010 (2)	−0.0054 (18)
C27	0.036 (2)	0.052 (3)	0.052 (3)	−0.010 (2)	−0.003 (2)	0.001 (2)
C28	0.025 (2)	0.059 (3)	0.061 (3)	0.006 (2)	0.004 (2)	0.009 (2)
C29	0.039 (3)	0.046 (3)	0.053 (3)	0.012 (2)	0.015 (2)	0.000 (2)
C30	0.037 (2)	0.036 (2)	0.038 (2)	0.0036 (18)	0.0033 (18)	0.0005 (18)
C31	0.032 (2)	0.035 (2)	0.036 (2)	0.0001 (17)	0.0041 (18)	0.0048 (17)
C32	0.042 (3)	0.049 (3)	0.053 (3)	0.013 (2)	0.007 (2)	0.014 (2)
C33	0.053 (3)	0.065 (3)	0.057 (3)	0.006 (3)	0.014 (2)	0.027 (2)
C34	0.060 (3)	0.050 (3)	0.067 (3)	0.008 (2)	−0.001 (3)	0.023 (2)
C35	0.054 (3)	0.045 (3)	0.065 (3)	0.021 (2)	0.004 (2)	0.009 (2)
C36	0.036 (2)	0.050 (3)	0.043 (2)	0.008 (2)	0.0061 (19)	0.001 (2)

### Geometric parameters (Å, °)

**Table:**

Fe1—C13	1.750 (4)	C7—H7	0.93
Fe1—C14	1.754 (4)	C8—H8A	0.97
Fe1—P1	2.2565 (10)	C8—H8B	0.97
Fe1—S1	2.2762 (11)	C9—H9A	0.97
Fe1—S2	2.2818 (11)	C9—H9B	0.97
Fe1—Fe2	2.5160 (8)	C15—C19	1.399 (5)
Fe2—C11	1.761 (5)	C15—C16	1.417 (6)
Fe2—C12	1.777 (5)	C15—H15	0.98
Fe2—C10	1.813 (5)	C16—C17	1.379 (6)
Fe2—S2	2.2546 (11)	C16—H16	0.98
Fe2—S1	2.2867 (11)	C17—C18	1.413 (6)
Fe3—C22	2.025 (4)	C17—H17	0.98
Fe3—C18	2.030 (4)	C18—C19	1.410 (5)
Fe3—C16	2.034 (4)	C18—H18	0.98
Fe3—C17	2.036 (4)	C19—H19	0.98
Fe3—C24	2.038 (4)	C20—C21	1.398 (5)
Fe3—C21	2.040 (4)	C20—C24	1.425 (5)
Fe3—C23	2.041 (3)	C20—H20	0.98
Fe3—C19	2.043 (4)	C21—C22	1.409 (5)
Fe3—C15	2.044 (4)	C21—H21	0.98
Fe3—C20	2.047 (4)	C22—C23	1.431 (4)
S1—C9	1.864 (3)	C22—H22	0.98
S2—C8	1.860 (4)	C23—C24	1.425 (4)
P1—C23	1.806 (3)	C24—H24	0.98
P1—C25	1.832 (3)	C25—C26	1.385 (4)
P1—C31	1.836 (3)	C25—C30	1.392 (4)
O1—C10	1.132 (4)	C26—C27	1.376 (5)
O2—C11	1.157 (4)	C26—H26	0.93
O3—C12	1.132 (4)	C27—C28	1.385 (5)
O4—C13	1.151 (4)	C27—H27	0.93
O5—C14	1.153 (4)	C28—C29	1.363 (5)
N1—C5	1.400 (4)	C28—H28	0.93
N1—C9	1.421 (4)	C29—C30	1.380 (5)
N1—C8	1.433 (4)	C29—H29	0.93
C1—C2	1.519 (5)	C30—H30	0.93
C1—H1A	0.96	C31—C36	1.382 (4)
C1—H1B	0.96	C31—C32	1.391 (4)
C1—H1C	0.96	C32—C33	1.386 (5)
C2—C3	1.376 (5)	C32—H32	0.93
C2—C7	1.385 (5)	C33—C34	1.363 (5)
C3—C4	1.374 (5)	C33—H33	0.93
C3—H3	0.93	C34—C35	1.364 (5)
C4—C5	1.396 (5)	C34—H34	0.93
C4—H4	0.93	C35—C36	1.386 (4)
C5—C6	1.384 (5)	C35—H35	0.93
C6—C7	1.381 (5)	C36—H36	0.93
C6—H6	0.93		
			
C13—Fe1—C14	91.41 (17)	N1—C8—S2	117.6 (2)
C13—Fe1—P1	96.67 (12)	N1—C8—H8A	107.9
C14—Fe1—P1	97.31 (12)	S2—C8—H8A	107.9
C13—Fe1—S1	87.66 (12)	N1—C8—H8B	107.9
C14—Fe1—S1	159.67 (12)	S2—C8—H8B	107.9
P1—Fe1—S1	102.97 (4)	H8A—C8—H8B	107.2
C13—Fe1—S2	152.46 (12)	N1—C9—S1	115.4 (2)
C14—Fe1—S2	87.77 (12)	N1—C9—H9A	108.4
P1—Fe1—S2	110.75 (4)	S1—C9—H9A	108.4
S1—Fe1—S2	83.80 (4)	N1—C9—H9B	108.4
C13—Fe1—Fe2	97.87 (12)	S1—C9—H9B	108.4
C14—Fe1—Fe2	103.39 (12)	H9A—C9—H9B	107.5
P1—Fe1—Fe2	154.30 (4)	O1—C10—Fe2	173.4 (4)
S1—Fe1—Fe2	56.73 (3)	O2—C11—Fe2	178.3 (4)
S2—Fe1—Fe2	55.80 (3)	O3—C12—Fe2	178.3 (4)
C11—Fe2—C12	90.8 (2)	O4—C13—Fe1	176.7 (3)
C11—Fe2—C10	100.0 (2)	O5—C14—Fe1	176.7 (4)
C12—Fe2—C10	95.26 (18)	C19—C15—C16	107.3 (5)
C11—Fe2—S2	159.16 (15)	C19—C15—Fe3	69.9 (3)
C12—Fe2—S2	89.71 (14)	C16—C15—Fe3	69.3 (3)
C10—Fe2—S2	100.70 (14)	C19—C15—H15	126.3
C11—Fe2—S1	85.74 (13)	C16—C15—H15	126.4
C12—Fe2—S1	152.32 (14)	Fe3—C15—H15	126.4
C10—Fe2—S1	112.39 (13)	C17—C16—C15	108.7 (5)
S2—Fe2—S1	84.18 (4)	C17—C16—Fe3	70.3 (3)
C11—Fe2—Fe1	102.51 (14)	C15—C16—Fe3	70.0 (3)
C12—Fe2—Fe1	97.99 (13)	C17—C16—H16	125.6
C10—Fe2—Fe1	153.62 (13)	C15—C16—H16	125.7
S2—Fe2—Fe1	56.83 (3)	Fe3—C16—H16	125.6
S1—Fe2—Fe1	56.34 (3)	C16—C17—C18	108.3 (5)
C22—Fe3—C18	164.24 (18)	C16—C17—Fe3	70.1 (3)
C22—Fe3—C16	120.53 (19)	C18—C17—Fe3	69.5 (2)
C18—Fe3—C16	67.69 (19)	C16—C17—H17	126.0
C22—Fe3—C17	153.86 (18)	C18—C17—H17	125.7
C18—Fe3—C17	40.67 (16)	Fe3—C17—H17	125.8
C16—Fe3—C17	39.61 (17)	C19—C18—C17	107.4 (4)
C22—Fe3—C24	68.59 (15)	C19—C18—Fe3	70.2 (2)
C18—Fe3—C24	108.54 (17)	C17—C18—Fe3	69.9 (2)
C16—Fe3—C24	159.7 (2)	C19—C18—H18	126.3
C17—Fe3—C24	124.54 (19)	C17—C18—H18	126.3
C22—Fe3—C21	40.55 (13)	Fe3—C18—H18	126.3
C18—Fe3—C21	154.22 (19)	C15—C19—C18	108.4 (4)
C16—Fe3—C21	106.06 (18)	C15—C19—Fe3	70.0 (2)
C17—Fe3—C21	118.59 (18)	C18—C19—Fe3	69.3 (2)
C24—Fe3—C21	68.17 (16)	C15—C19—H19	125.9
C22—Fe3—C23	41.20 (12)	C18—C19—H19	125.8
C18—Fe3—C23	126.57 (17)	Fe3—C19—H19	126.0
C16—Fe3—C23	157.2 (2)	C21—C20—C24	108.1 (4)
C17—Fe3—C23	162.63 (19)	C21—C20—Fe3	69.8 (2)
C24—Fe3—C23	40.89 (12)	C24—C20—Fe3	69.3 (2)
C21—Fe3—C23	68.77 (14)	C21—C20—H20	125.8
C22—Fe3—C19	127.14 (18)	C24—C20—H20	126.0
C18—Fe3—C19	40.50 (15)	Fe3—C20—H20	126.1
C16—Fe3—C19	67.59 (18)	C20—C21—C22	108.5 (4)
C17—Fe3—C19	67.78 (18)	C20—C21—Fe3	70.2 (2)
C24—Fe3—C19	123.45 (18)	C22—C21—Fe3	69.1 (2)
C21—Fe3—C19	162.4 (2)	C20—C21—H21	125.9
C23—Fe3—C19	110.29 (16)	C22—C21—H21	125.6
C22—Fe3—C15	108.63 (18)	Fe3—C21—H21	125.7
C18—Fe3—C15	67.97 (19)	C21—C22—C23	108.5 (3)
C16—Fe3—C15	40.67 (16)	C21—C22—Fe3	70.3 (2)
C17—Fe3—C15	67.7 (2)	C23—C22—Fe3	70.00 (19)
C24—Fe3—C15	158.38 (19)	C21—C22—H22	125.9
C21—Fe3—C15	124.6 (2)	C23—C22—H22	125.6
C23—Fe3—C15	122.80 (18)	Fe3—C22—H22	125.7
C19—Fe3—C15	40.03 (16)	C24—C23—C22	106.6 (3)
C22—Fe3—C20	68.06 (16)	C24—C23—P1	126.1 (3)
C18—Fe3—C20	120.63 (19)	C22—C23—P1	126.8 (3)
C16—Fe3—C20	122.40 (18)	C24—C23—Fe3	69.4 (2)
C17—Fe3—C20	106.01 (18)	C22—C23—Fe3	68.8 (2)
C24—Fe3—C20	40.84 (13)	P1—C23—Fe3	132.96 (18)
C21—Fe3—C20	40.00 (14)	C23—C24—C20	108.2 (3)
C23—Fe3—C20	68.76 (14)	C23—C24—Fe3	69.7 (2)
C19—Fe3—C20	157.4 (2)	C20—C24—Fe3	69.9 (2)
C15—Fe3—C20	159.8 (2)	C23—C24—H24	125.9
C9—S1—Fe1	107.79 (13)	C20—C24—H24	125.9
C9—S1—Fe2	111.20 (12)	Fe3—C24—H24	125.9
Fe1—S1—Fe2	66.93 (3)	C26—C25—C30	118.0 (3)
C8—S2—Fe2	108.94 (12)	C26—C25—P1	118.5 (3)
C8—S2—Fe1	116.93 (13)	C30—C25—P1	123.4 (3)
Fe2—S2—Fe1	67.37 (3)	C27—C26—C25	121.5 (3)
C23—P1—C25	102.96 (16)	C27—C26—H26	119.3
C23—P1—C31	104.03 (16)	C25—C26—H26	119.3
C25—P1—C31	100.01 (15)	C26—C27—C28	119.6 (4)
C23—P1—Fe1	118.32 (12)	C26—C27—H27	120.2
C25—P1—Fe1	112.95 (11)	C28—C27—H27	120.2
C31—P1—Fe1	116.22 (12)	C29—C28—C27	119.7 (4)
C5—N1—C9	119.7 (3)	C29—C28—H28	120.2
C5—N1—C8	123.4 (3)	C27—C28—H28	120.2
C9—N1—C8	112.7 (3)	C28—C29—C30	120.9 (4)
C2—C1—H1A	109.5	C28—C29—H29	119.5
C2—C1—H1B	109.5	C30—C29—H29	119.5
H1A—C1—H1B	109.5	C29—C30—C25	120.3 (4)
C2—C1—H1C	109.5	C29—C30—H30	119.9
H1A—C1—H1C	109.5	C25—C30—H30	119.9
H1B—C1—H1C	109.5	C36—C31—C32	117.4 (3)
C3—C2—C7	116.6 (4)	C36—C31—P1	121.7 (3)
C3—C2—C1	122.2 (4)	C32—C31—P1	120.8 (3)
C7—C2—C1	121.2 (4)	C33—C32—C31	121.1 (4)
C4—C3—C2	122.7 (4)	C33—C32—H32	119.4
C4—C3—H3	118.7	C31—C32—H32	119.4
C2—C3—H3	118.7	C34—C33—C32	120.0 (4)
C3—C4—C5	120.6 (4)	C34—C33—H33	120.0
C3—C4—H4	119.7	C32—C33—H33	120.0
C5—C4—H4	119.7	C33—C34—C35	120.3 (4)
C6—C5—C4	117.1 (4)	C33—C34—H34	119.9
C6—C5—N1	122.2 (4)	C35—C34—H34	119.9
C4—C5—N1	120.6 (4)	C34—C35—C36	120.0 (4)
C7—C6—C5	121.3 (4)	C34—C35—H35	120.0
C7—C6—H6	119.4	C36—C35—H35	120.0
C5—C6—H6	119.4	C31—C36—C35	121.3 (4)
C6—C7—C2	121.7 (4)	C31—C36—H36	119.4
C6—C7—H7	119.1	C35—C36—H36	119.4
C2—C7—H7	119.1		
			
C13—Fe1—Fe2—C11	−5.79 (19)	C20—Fe3—C18—C17	−78.6 (3)
C14—Fe1—Fe2—C11	−99.1 (2)	C16—C15—C19—C18	0.7 (5)
P1—Fe1—Fe2—C11	118.10 (17)	Fe3—C15—C19—C18	−58.8 (3)
S1—Fe1—Fe2—C11	76.16 (16)	C16—C15—C19—Fe3	59.5 (3)
S2—Fe1—Fe2—C11	−177.00 (16)	C17—C18—C19—C15	−1.0 (5)
C13—Fe1—Fe2—C12	86.84 (18)	Fe3—C18—C19—C15	59.3 (3)
C14—Fe1—Fe2—C12	−6.51 (19)	C17—C18—C19—Fe3	−60.3 (3)
P1—Fe1—Fe2—C12	−149.28 (16)	C22—Fe3—C19—C15	73.8 (3)
S1—Fe1—Fe2—C12	168.78 (14)	C18—Fe3—C19—C15	−119.7 (4)
S2—Fe1—Fe2—C12	−84.38 (14)	C16—Fe3—C19—C15	−38.3 (3)
C13—Fe1—Fe2—C10	−153.7 (3)	C17—Fe3—C19—C15	−81.3 (3)
C14—Fe1—Fe2—C10	112.9 (3)	C24—Fe3—C19—C15	161.0 (3)
P1—Fe1—Fe2—C10	−29.8 (3)	C21—Fe3—C19—C15	33.6 (7)
S1—Fe1—Fe2—C10	−71.8 (3)	C23—Fe3—C19—C15	117.2 (3)
S2—Fe1—Fe2—C10	35.1 (3)	C20—Fe3—C19—C15	−159.5 (4)
C13—Fe1—Fe2—S2	171.21 (12)	C22—Fe3—C19—C18	−166.5 (3)
C14—Fe1—Fe2—S2	77.86 (13)	C16—Fe3—C19—C18	81.4 (3)
P1—Fe1—Fe2—S2	−64.90 (8)	C17—Fe3—C19—C18	38.4 (3)
S1—Fe1—Fe2—S2	−106.84 (4)	C24—Fe3—C19—C18	−79.2 (3)
C13—Fe1—Fe2—S1	−81.95 (12)	C21—Fe3—C19—C18	153.3 (5)
C14—Fe1—Fe2—S1	−175.30 (13)	C23—Fe3—C19—C18	−123.0 (3)
P1—Fe1—Fe2—S1	41.94 (8)	C15—Fe3—C19—C18	119.7 (4)
S2—Fe1—Fe2—S1	106.84 (4)	C20—Fe3—C19—C18	−39.7 (6)
C13—Fe1—S1—C9	−152.98 (16)	C22—Fe3—C20—C21	−37.4 (2)
C14—Fe1—S1—C9	119.3 (4)	C18—Fe3—C20—C21	157.3 (3)
P1—Fe1—S1—C9	−56.67 (12)	C16—Fe3—C20—C21	75.7 (3)
S2—Fe1—S1—C9	53.25 (12)	C17—Fe3—C20—C21	115.6 (3)
Fe2—Fe1—S1—C9	106.02 (12)	C24—Fe3—C20—C21	−119.6 (3)
C13—Fe1—S1—Fe2	101.00 (11)	C23—Fe3—C20—C21	−81.9 (2)
C14—Fe1—S1—Fe2	13.3 (4)	C19—Fe3—C20—C21	−173.9 (4)
P1—Fe1—S1—Fe2	−162.70 (4)	C15—Fe3—C20—C21	47.0 (6)
S2—Fe1—S1—Fe2	−52.78 (3)	C22—Fe3—C20—C24	82.2 (2)
C11—Fe2—S1—C9	150.9 (2)	C18—Fe3—C20—C24	−83.1 (3)
C12—Fe2—S1—C9	−125.5 (3)	C16—Fe3—C20—C24	−164.7 (3)
C10—Fe2—S1—C9	51.8 (2)	C17—Fe3—C20—C24	−124.8 (3)
S2—Fe2—S1—C9	−47.37 (13)	C21—Fe3—C20—C24	119.6 (3)
Fe1—Fe2—S1—C9	−101.01 (13)	C23—Fe3—C20—C24	37.7 (2)
C11—Fe2—S1—Fe1	−108.09 (16)	C19—Fe3—C20—C24	−54.3 (5)
C12—Fe2—S1—Fe1	−24.5 (3)	C15—Fe3—C20—C24	166.6 (5)
C10—Fe2—S1—Fe1	152.84 (15)	C24—C20—C21—C22	−0.1 (5)
S2—Fe2—S1—Fe1	53.64 (3)	Fe3—C20—C21—C22	58.7 (3)
C11—Fe2—S2—C8	120.3 (4)	C24—C20—C21—Fe3	−58.8 (3)
C12—Fe2—S2—C8	−148.17 (19)	C22—Fe3—C21—C20	119.9 (4)
C10—Fe2—S2—C8	−52.87 (18)	C18—Fe3—C21—C20	−49.8 (5)
S1—Fe2—S2—C8	58.87 (13)	C16—Fe3—C21—C20	−121.6 (3)
Fe1—Fe2—S2—C8	112.07 (13)	C17—Fe3—C21—C20	−80.8 (3)
C11—Fe2—S2—Fe1	8.2 (4)	C24—Fe3—C21—C20	37.8 (2)
C12—Fe2—S2—Fe1	99.76 (14)	C23—Fe3—C21—C20	81.9 (2)
C10—Fe2—S2—Fe1	−164.94 (13)	C19—Fe3—C21—C20	172.2 (5)
S1—Fe2—S2—Fe1	−53.20 (3)	C15—Fe3—C21—C20	−162.2 (3)
C13—Fe1—S2—C8	−119.6 (3)	C18—Fe3—C21—C22	−169.7 (4)
C14—Fe1—S2—C8	151.61 (18)	C16—Fe3—C21—C22	118.5 (3)
P1—Fe1—S2—C8	54.64 (14)	C17—Fe3—C21—C22	159.3 (3)
S1—Fe1—S2—C8	−46.91 (14)	C24—Fe3—C21—C22	−82.1 (3)
Fe2—Fe1—S2—C8	−100.52 (14)	C23—Fe3—C21—C22	−38.0 (2)
C13—Fe1—S2—Fe2	−19.1 (3)	C19—Fe3—C21—C22	52.3 (7)
C14—Fe1—S2—Fe2	−107.87 (12)	C15—Fe3—C21—C22	77.9 (3)
P1—Fe1—S2—Fe2	155.17 (4)	C20—Fe3—C21—C22	−119.9 (4)
S1—Fe1—S2—Fe2	53.61 (3)	C20—C21—C22—C23	0.4 (5)
C13—Fe1—P1—C23	−153.80 (18)	Fe3—C21—C22—C23	59.8 (3)
C14—Fe1—P1—C23	−61.51 (18)	C20—C21—C22—Fe3	−59.4 (3)
S1—Fe1—P1—C23	117.07 (13)	C18—Fe3—C22—C21	163.4 (6)
S2—Fe1—P1—C23	28.86 (14)	C16—Fe3—C22—C21	−78.7 (3)
Fe2—Fe1—P1—C23	82.08 (15)	C17—Fe3—C22—C21	−44.7 (5)
C13—Fe1—P1—C25	85.87 (17)	C24—Fe3—C22—C21	81.0 (3)
C14—Fe1—P1—C25	178.16 (17)	C23—Fe3—C22—C21	119.3 (3)
S1—Fe1—P1—C25	−3.25 (13)	C19—Fe3—C22—C21	−162.5 (3)
S2—Fe1—P1—C25	−91.46 (13)	C15—Fe3—C22—C21	−121.8 (3)
Fe2—Fe1—P1—C25	−38.24 (16)	C20—Fe3—C22—C21	36.9 (2)
C13—Fe1—P1—C31	−28.89 (18)	C18—Fe3—C22—C23	44.1 (7)
C14—Fe1—P1—C31	63.40 (18)	C16—Fe3—C22—C23	162.0 (2)
S1—Fe1—P1—C31	−118.01 (13)	C17—Fe3—C22—C23	−164.0 (4)
S2—Fe1—P1—C31	153.78 (13)	C24—Fe3—C22—C23	−38.3 (2)
Fe2—Fe1—P1—C31	−153.00 (13)	C21—Fe3—C22—C23	−119.3 (3)
C7—C2—C3—C4	0.1 (6)	C19—Fe3—C22—C23	78.2 (3)
C1—C2—C3—C4	179.4 (4)	C15—Fe3—C22—C23	118.9 (3)
C2—C3—C4—C5	−0.9 (6)	C20—Fe3—C22—C23	−82.4 (2)
C3—C4—C5—C6	1.4 (5)	C21—C22—C23—C24	−0.6 (4)
C3—C4—C5—N1	177.6 (3)	Fe3—C22—C23—C24	59.4 (2)
C9—N1—C5—C6	3.7 (5)	C21—C22—C23—P1	171.5 (3)
C8—N1—C5—C6	−151.7 (3)	Fe3—C22—C23—P1	−128.5 (3)
C9—N1—C5—C4	−172.3 (3)	C21—C22—C23—Fe3	−60.0 (3)
C8—N1—C5—C4	32.3 (5)	C25—P1—C23—C24	109.2 (3)
C4—C5—C6—C7	−1.1 (6)	C31—P1—C23—C24	−146.8 (3)
N1—C5—C6—C7	−177.2 (3)	Fe1—P1—C23—C24	−16.1 (4)
C5—C6—C7—C2	0.3 (6)	C25—P1—C23—C22	−61.3 (3)
C3—C2—C7—C6	0.2 (6)	C31—P1—C23—C22	42.6 (4)
C1—C2—C7—C6	−179.0 (4)	Fe1—P1—C23—C22	173.3 (3)
C5—N1—C8—S2	98.7 (4)	C25—P1—C23—Fe3	−155.9 (2)
C9—N1—C8—S2	−58.1 (4)	C31—P1—C23—Fe3	−51.9 (3)
Fe2—S2—C8—N1	−20.0 (3)	Fe1—P1—C23—Fe3	78.7 (3)
Fe1—S2—C8—N1	53.6 (3)	C22—Fe3—C23—C24	−118.2 (3)
C5—N1—C9—S1	−84.2 (4)	C18—Fe3—C23—C24	75.4 (3)
C8—N1—C9—S1	73.6 (3)	C16—Fe3—C23—C24	−161.7 (4)
Fe1—S1—C9—N1	−79.0 (3)	C17—Fe3—C23—C24	37.8 (6)
Fe2—S1—C9—N1	−7.5 (3)	C21—Fe3—C23—C24	−80.8 (2)
C22—Fe3—C15—C19	−126.1 (3)	C19—Fe3—C23—C24	118.1 (2)
C18—Fe3—C15—C19	37.5 (3)	C15—Fe3—C23—C24	161.0 (2)
C16—Fe3—C15—C19	118.4 (4)	C20—Fe3—C23—C24	−37.7 (2)
C17—Fe3—C15—C19	81.6 (3)	C18—Fe3—C23—C22	−166.4 (3)
C24—Fe3—C15—C19	−47.4 (6)	C16—Fe3—C23—C22	−43.4 (5)
C21—Fe3—C15—C19	−168.2 (3)	C17—Fe3—C23—C22	156.0 (5)
C23—Fe3—C15—C19	−82.8 (3)	C24—Fe3—C23—C22	118.2 (3)
C20—Fe3—C15—C19	157.0 (5)	C21—Fe3—C23—C22	37.5 (2)
C22—Fe3—C15—C16	115.5 (3)	C19—Fe3—C23—C22	−123.7 (3)
C18—Fe3—C15—C16	−80.9 (3)	C15—Fe3—C23—C22	−80.8 (3)
C17—Fe3—C15—C16	−36.9 (3)	C20—Fe3—C23—C22	80.5 (2)
C24—Fe3—C15—C16	−165.8 (4)	C22—Fe3—C23—P1	121.1 (4)
C21—Fe3—C15—C16	73.3 (4)	C18—Fe3—C23—P1	−45.3 (4)
C23—Fe3—C15—C16	158.8 (3)	C16—Fe3—C23—P1	77.7 (5)
C19—Fe3—C15—C16	−118.4 (4)	C17—Fe3—C23—P1	−82.9 (6)
C20—Fe3—C15—C16	38.6 (7)	C24—Fe3—C23—P1	−120.7 (3)
C19—C15—C16—C17	−0.1 (5)	C21—Fe3—C23—P1	158.6 (3)
Fe3—C15—C16—C17	59.8 (3)	C19—Fe3—C23—P1	−2.6 (3)
C19—C15—C16—Fe3	−59.9 (3)	C15—Fe3—C23—P1	40.3 (3)
C22—Fe3—C16—C17	157.2 (3)	C20—Fe3—C23—P1	−158.4 (3)
C18—Fe3—C16—C17	−37.8 (3)	C22—C23—C24—C20	0.5 (4)
C24—Fe3—C16—C17	45.4 (6)	P1—C23—C24—C20	−171.6 (3)
C21—Fe3—C16—C17	115.7 (3)	Fe3—C23—C24—C20	59.5 (3)
C23—Fe3—C16—C17	−171.0 (3)	C22—C23—C24—Fe3	−59.0 (2)
C19—Fe3—C16—C17	−81.8 (3)	P1—C23—C24—Fe3	128.9 (3)
C15—Fe3—C16—C17	−119.5 (4)	C21—C20—C24—C23	−0.2 (4)
C20—Fe3—C16—C17	75.3 (4)	Fe3—C20—C24—C23	−59.4 (3)
C22—Fe3—C16—C15	−83.3 (3)	C21—C20—C24—Fe3	59.1 (3)
C18—Fe3—C16—C15	81.7 (3)	C22—Fe3—C24—C23	38.56 (19)
C17—Fe3—C16—C15	119.5 (4)	C18—Fe3—C24—C23	−124.9 (2)
C24—Fe3—C16—C15	164.9 (4)	C16—Fe3—C24—C23	159.4 (5)
C21—Fe3—C16—C15	−124.8 (3)	C17—Fe3—C24—C23	−167.2 (2)
C23—Fe3—C16—C15	−51.5 (6)	C21—Fe3—C24—C23	82.3 (2)
C19—Fe3—C16—C15	37.7 (3)	C19—Fe3—C24—C23	−82.6 (3)
C20—Fe3—C16—C15	−165.2 (3)	C15—Fe3—C24—C23	−48.1 (5)
C15—C16—C17—C18	−0.6 (5)	C20—Fe3—C24—C23	119.3 (3)
Fe3—C16—C17—C18	59.1 (3)	C22—Fe3—C24—C20	−80.8 (2)
C15—C16—C17—Fe3	−59.7 (3)	C18—Fe3—C24—C20	115.7 (3)
C22—Fe3—C17—C16	−49.2 (6)	C16—Fe3—C24—C20	40.1 (6)
C18—Fe3—C17—C16	119.5 (5)	C17—Fe3—C24—C20	73.5 (3)
C24—Fe3—C17—C16	−162.6 (3)	C21—Fe3—C24—C20	−37.0 (2)
C21—Fe3—C17—C16	−80.5 (4)	C23—Fe3—C24—C20	−119.3 (3)
C23—Fe3—C17—C16	168.3 (5)	C19—Fe3—C24—C20	158.0 (3)
C19—Fe3—C17—C16	81.2 (3)	C15—Fe3—C24—C20	−167.4 (5)
C15—Fe3—C17—C16	37.8 (3)	C23—P1—C25—C26	176.8 (3)
C20—Fe3—C17—C16	−121.8 (3)	C31—P1—C25—C26	69.7 (3)
C22—Fe3—C17—C18	−168.7 (4)	Fe1—P1—C25—C26	−54.5 (3)
C16—Fe3—C17—C18	−119.5 (5)	C23—P1—C25—C30	−6.0 (3)
C24—Fe3—C17—C18	77.9 (3)	C31—P1—C25—C30	−113.1 (3)
C21—Fe3—C17—C18	160.0 (3)	Fe1—P1—C25—C30	122.8 (3)
C23—Fe3—C17—C18	48.8 (7)	C30—C25—C26—C27	−0.3 (5)
C19—Fe3—C17—C18	−38.3 (3)	P1—C25—C26—C27	177.1 (3)
C15—Fe3—C17—C18	−81.7 (3)	C25—C26—C27—C28	0.1 (6)
C20—Fe3—C17—C18	118.6 (3)	C26—C27—C28—C29	1.2 (6)
C16—C17—C18—C19	1.0 (5)	C27—C28—C29—C30	−2.2 (6)
Fe3—C17—C18—C19	60.5 (3)	C28—C29—C30—C25	2.1 (6)
C16—C17—C18—Fe3	−59.6 (3)	C26—C25—C30—C29	−0.8 (5)
C22—Fe3—C18—C19	43.4 (8)	P1—C25—C30—C29	−178.0 (3)
C16—Fe3—C18—C19	−81.1 (3)	C23—P1—C31—C36	104.2 (3)
C17—Fe3—C18—C19	−118.0 (4)	C25—P1—C31—C36	−149.6 (3)
C24—Fe3—C18—C19	120.2 (3)	Fe1—P1—C31—C36	−27.7 (3)
C21—Fe3—C18—C19	−161.8 (4)	C23—P1—C31—C32	−74.4 (3)
C23—Fe3—C18—C19	78.3 (3)	C25—P1—C31—C32	31.8 (3)
C15—Fe3—C18—C19	−37.1 (3)	Fe1—P1—C31—C32	153.6 (3)
C20—Fe3—C18—C19	163.4 (3)	C36—C31—C32—C33	−0.8 (6)
C22—Fe3—C18—C17	161.4 (6)	P1—C31—C32—C33	177.8 (3)
C16—Fe3—C18—C17	36.9 (3)	C31—C32—C33—C34	0.9 (6)
C24—Fe3—C18—C17	−121.8 (3)	C32—C33—C34—C35	−0.2 (7)
C21—Fe3—C18—C17	−43.8 (6)	C33—C34—C35—C36	−0.4 (7)
C23—Fe3—C18—C17	−163.8 (3)	C32—C31—C36—C35	0.2 (6)
C19—Fe3—C18—C17	118.0 (4)	P1—C31—C36—C35	−178.5 (3)
C15—Fe3—C18—C17	80.9 (3)	C34—C35—C36—C31	0.4 (6)

### Hydrogen-bond geometry (Å, °)

**Table:**

*D*—H···*A*	*D*—H	H···*A*	*D*···*A*	*D*—H···*A*
C22—H22···O2^i^	0.98	2.53	3.224 (5)	128
C24—H24···S2	0.98	2.72	3.561 (4)	144

Symmetry codes: (i) *x*, −*y*+1/2, *z*−1/2.
